# Geospatial distribution and associated factors of cancer cases registered at a tertiary hospital in Northwestern Tanzania. A retrospective study

**DOI:** 10.3389/fonc.2026.1859286

**Published:** 2026-07-22

**Authors:** Alen A. Kanjanja, Titus R. Leeyio, Nestory Masalu, Elias C. Nyanza, Kristin Schroeder

**Affiliations:** 1 Department of Environmental and Occupational Health, School of Public Health, Catholic University of Health and Allied Sciences, Mwanza, Tanzania; 2 Department of Obstretic and Gynercology, Sekou Toure Regional Referral Hospital, Mwanza, Tanzania; 3 Department of Epidemiology, Biostatistics and Behavioral Sciences, School of Public Health, Catholic University of Health and Allied Sciences, Mwanza, Tanzania; 4 Department of Oncology, Bugando Medical Center, Mwanza, Tanzania; 5 Pediatric Oncology and Global Health, Duke University Durham, Durham, NC, United States

**Keywords:** cancer, cervical uteri, geographical patterns, malignant neoplasm, prostate cancer

## Abstract

**Background:**

Cancer continues to be ranked high in causing mortality and morbidity among non-communicable diseases, being the second to cardiovascular diseases with 10 million deaths annually. This retrospective longitudinal hospital-based study evaluated the records of 3697 cancer patients from January 2019 to December 2021 at Bugando Medical Centre (BMC), a tertiary referral hospital in northwestern Tanzania.

**Methods:**

We employed a retrospective longitudinal study of cancer patients at BMC (2019–2021) using Mwanza Cancer Registry (MCR) data. Sociodemographic and clinical data were extracted and partially validated. Analyses in Stata v18 included descriptive statistics and modified Poisson regression. Geographic distribution was mapped using QGIS, with clustering assessed via Ripley’s K. Ethical approval and confidentiality were maintained.

**Results:**

The Median age was 53 years (IQR: 39-65). The Majority (22%) of the participants were diagnosed with malignant neoplasm of cervix cancer. About (11.2%) of the participants were diagnosed with malignant neoplasm of the prostate. Participants aged 52–68 years had a 54% higher proportion of diagnosed cervix cancer cancer cases (aPR= 1.54, 95% CI = 1.21- 1.78). Participants aged 69–98 years had a 75% higher proportion of diagnosed cervix cancer cancer cases (aPR= 1.75, 95% CI = 1.12- 1.99). Some cancer hotspots prevail in different parts of the Lake Zone.

**Conclusion:**

Diagnosed cancer cases in the Lake Zone are unevenly distributed, with some areas presenting more diagnosed patients as compared to others. There are vivid hotspots for different cancer types in different areas of the Lake Zone that can be used for cancer programs in the region. Cervix cancer and prostate cancer were the most common among all patients diagnosed at BMC in the last three years. The burden of cervical cancer is high in the Lake Zone.

## 1 Introduction

Cancer is a major public health challenge and one of the leading causes of morbidity and mortality worldwide. In 2022, an estimated 20 million new cancer cases and 9.7 million cancer-related deaths occurred globally and this burden is projected to increase substantially over the coming decades due to population growth, ageing and continuous exposure to cancer risk factors (1–3). More than half of the newly diagnosed cancers and nearly 70% of cancer-related deaths occur in low-and middle-income countries (LMICs), where limited access to prevention, early detection, diagnosis and treatment services contribute to poor outcomes (1).

Overall, breast cancer accounts for 11.7% of the total reported cases, followed by lung (11.4%), colorectal (10.0%), prostate (7.3%), and stomach (5.6%) cancers. Lung cancer has the highest associated mortality, responsible for 18% of cancer-related deaths globally (3). Epidemiological reports on cancer burden show a large heterogeneity by region, reflecting variation in potential environmental exposure, social economics, and access to medical care and screening (4, 5). Therefore, medical geography presents a potential cornerstone analysis to review the relationship between the environment and disease (6–8). Currently, 57% of all new cancers and more than 70% of cancer-related deaths around the globe are reported to occur in LMICs (9, 10).

Tanzania continues to experience an increasing number of cancer diagnoses with cervical, breast, prostate, esophageal cancer and Kaposi sarcoma being the most commonly reported malignancies. According to the Tanzania 2013–2022 Cancer Control Strategy (CCS), it is estimated that over 40,000 new cases are being reported yearly (11). The mortality rate reported to be around 68% in 2021 (12, 13). Moreover, the common causes of death in Tanzania’s hospitals showed that cancer was the 7^th^ leading cause of death, contributing to slightly above 5% of all deaths occurring in hospitals among individuals aged 15–59 years (14, 15). Despite ongoing efforts to strengthen cancer prevention and control programs, many patients are diagnosed at advanced stages of disease, resulting in substantial morbidity and mortality. Reliable epidemiological and geographical information is therefore essential for guiding resource allocation, planning and improving cancer services. Spatial epidemiology and geographic information system (GIS) have increasingly been used to examine the distribution of cancer cases, identify areas with disproportionately high disease burden and generate hypotheses. These have been used to inform on the environmental, demographic and healthcare-related factors that may influence cancer occurrences. Such information is valuable in resource-limited settings such as the Lake Zone in Tanzania where health resources must be strategically allocated.

The Lake Zone has the second-highest cancer-related mortality and morbidity in Tanzania (14, 16). Cancer diagnosis in the Lake Zone have shown considerable geographic variation across districts and regions suggesting differences in environmental exposures, population characteristics, healthcare access and cancer detection practices (2). Published studies examined and described cancer burden and trends in Tanzania and along the lake. Specifically, limited evidence exists regarding the spatial distribution of cancer cases and cancer-specific hotspots using registry-derived data. Therefore, this study aimed to describe the geospatial distribution of cancer cases registered at Bugando Medical Centre (BMC) and to identify demographic and geographic factors associated with the most common cancer diagnosed between 2019 and 2021. The findings will support evidence-based cancer control strategies and guide the prioritization of interventions in high-burden areas of the Lake Zone and in Tanzania.

## 2 Methods

### 2.1 Study design, and study settings

This was a retrospective, longitudinal hospital-based study among cancer patients attending BMC. The details of the study settings have been explained elsewhere (15). Briefly, BMC is a tertiary care and teaching hospital located in Northwestern Tanzania near the shores of Lake Victoria. The hospital currently serves a catchment area population of approximately 20 million people from the Lake Zones: Mwanza, Geita, Kagera, Mara, Simiyu, Shinyanga, Tabora, and Kigoma (17). Bugando Medical Centre has specialized medical services in all major disciplines of medicine with a bed capacity of more than 1,000 beds (11). The Oncology Department attends an average of 60 patients per day in all its clinics. The department provides chemotherapy, radiotherapy treatment, and palliative care.

### 2.2 Ethics considerations

The study protocol was approved by the Joint Catholic University of Health and Allied Sciences and Bugando Medical Centre (CREC#570/2022), with additional permission from BMC on data access. Verbal informed consent was obtained only from participants contracted for residential validation. To ensure confidentiality, all data were de-identified using unique codes and stored securely in password-protected systems and locked cabinets, accessible only to authorized study personnel.

### 2.3 Study population and data collection

The study used archived patient records in the Mwanza Cancer Registry (MCR) which is one of the three cancer registries covering the Lake Zone situated at BMC (16). All retrieved records were recorded from January 2019 to December 2021. The Lake Zone is grouped into three cluster/zones: the mining zone with exposure to heavy metals, the fishing zone with endemic schistosomiasis, and agro-based areas prone to aflatoxin contamination found in nuts (18–20). Following its establishment in 2016, the MCR recorded a total of 1,500 cancer cases over a four-year period (2016–2019) (21, 22).

The study population included all inpatients and outpatients of all ages and sex who visited the BMC Oncology Department and were registered in the MCR during the study period. They included patients with a laboratory, imaging, pathological (biopsy), or clinically confirmed diagnosis of cancer between January 1^st^, 2019, and December 31^st^, 2021. A validated data collection checklist was used to extract information from MCR. Extracted data included socio-demographic information (age, gender, ethnic group, area of residence), date of final diagnosis, diagnosis status and patient outcome. A random sample of patients with reachable phone numbers in the MCR were called to validate their residential status (75% of records were validated).

### 2.4 Statistical analysis

STATA version 18 (Stata Corp Station, TX, USA) was used for data cleaning and analysis. Some variables were combined to increase the power of the groups. Continuous variables were summarized using medians and interquartile ranges (IQRs) or means and standard deviations (SDs) while categorical variables were summarized using frequencies and proportions. Separate binary outcomes were created for each of the major cancer types including cervical, prostate, breast, head and neck, esophageal cancer and non-Hodgkin lymphoma. Associations between independent variables such as demographics and geographic factors and each outcome were assessed using modified Poisson regression with robust standard errors to estimate crude and adjusted prevalence ratios (aPR) with 95% confidence intervals to determine significance.

Modified Poisson regression was selected because several cancer outcomes were relatively common within the study population, making prevalence ratios more interpretable than odds ratios and avoiding overestimation associated with logistic regression when outcomes are not rare. Variables with *p<0.20*, together with those identified *a priori* based on existing literature and biological plausibility, were included in the multivariable models to control for potential confounding.

Separate logistic regression models were fitted for different cancer outcomes. For breast cancer, head and neck cancer, esophageal cancer and non-Hodgkin lymphoma, binary logistic regression models were fitted and the results were reported as odds ratio (ORs). Logistic regression was used because these outcomes had lower frequencies and demonstrated stable model convergence. However, because some outcomes were not rare, the reported ORs should not be interpreted as direct approximations of the prevalence ratio.

Multivariable regression models were constructed without the use of automated stepwise procedures. Confounding was assessed using a change-in-estimate approach, whereby variables were retained if their inclusion altered the main association by ≥10%, regardless of statistical significance. Statistical significance was assessed using 95% confidence intervals as well as a cutoff of *p <0.05*.

### 2.5 Geographical distribution of analysis of cancer cases

The recorded residences for patients from the MCR or archived patients’ files were tracked using Google Maps and Geographical Information System (GIS) to capture the location of registered villages and/or streets to conduct a descriptive mapping. Where available, coordinates were assigned at the village or ward level. For records lacking sufficient detail for precise geocoding, cases were aggregated to the district level and records with incomplete or unverifiable residential information were excluded from point-level spatial analyses. The primary unit of analysis was the district where cancer case counts were aggregated by district and normalized using district population estimates obtained from the 2022 Tanzania Population and Housing Census Projections (12, 13). Maps were produced using Quantum Geographical Information System (QGIS) *software version 3.44.7*. Choropleth maps were generated to illustrate the geographic distribution of cancer cases and cancer-specific burden across the Lake Zone. Areas with comparatively high case burdens were considered hotspots for descriptive purposes. The QGIS software was used to transfer the data into the map and the spatial distribution of cancers was produced. To evaluate spatial clustering of geocoded cancer cases, Ripley’s K-function was applied using point-level coordinate data and compared with the expected distribution under complete spatial randomness (CSR) (13, 14, 23). Hotspot identification was based on observed concentrations of normalized cancer cases rather than formal cluster detection algorithms. No kernel density estimation or spatial smoothing procedures were applied. Similarly, spatial autocorrelation statistics such as Moran’s I were not performed because the objective was descriptive mapping and exploratory cluster assessment using the Ripley’s K-function.

## 3 Results

### 3.1 Background characteristics of the study participants and their geographical distribution

**Figure 1:**
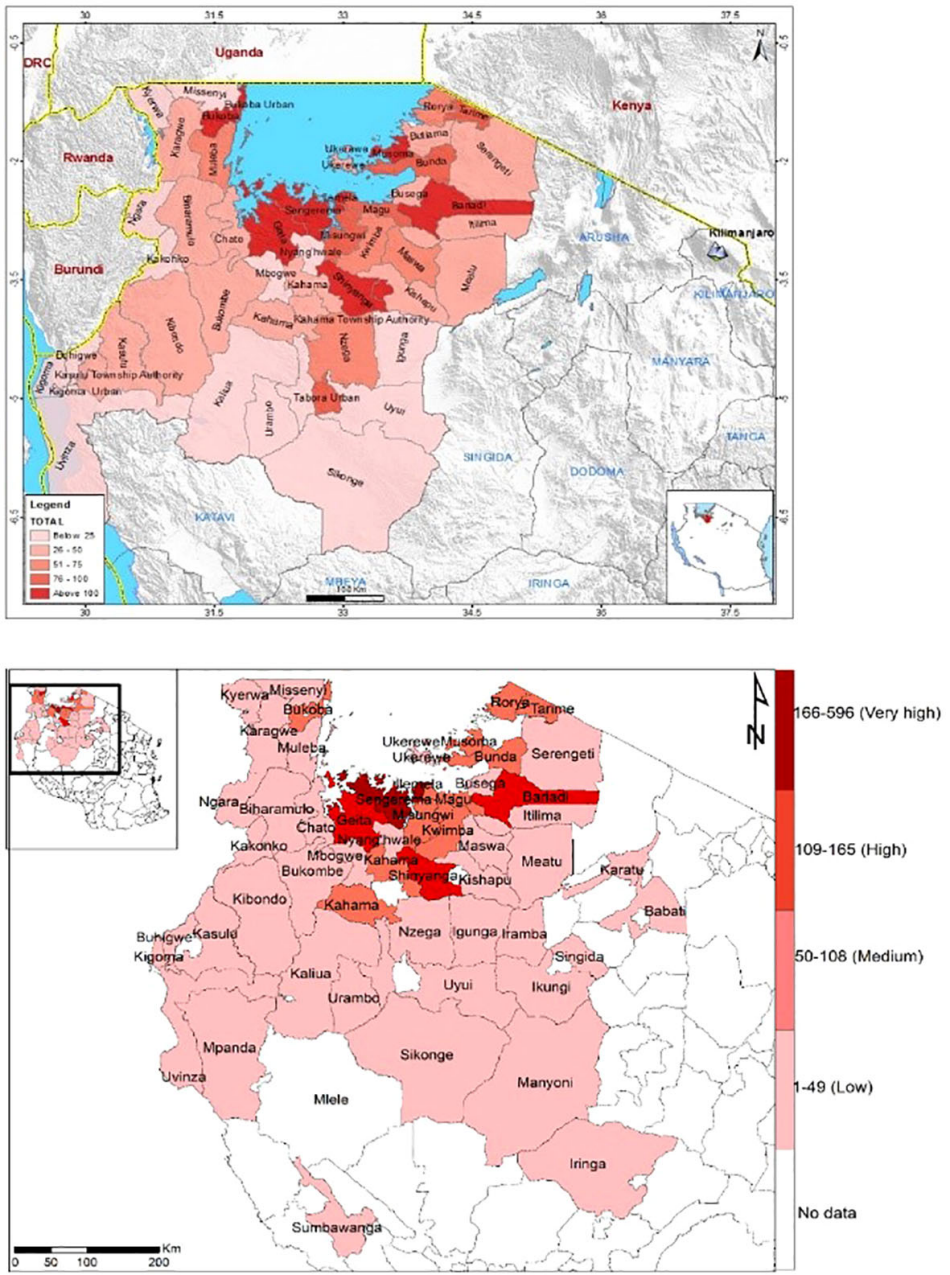
Geographical distribution of total cancer cases attended [Jan 2019 – Dec 2021 (N = 3697)]. Adapted from (24).

A total of 3697 participants were included in the analysis. From **Table 1**, about 34.5% of the participants were aged between 52 and 68 years. Their median age was 53(IQR: 39-65) years. More than half (55.7%) of the participants were female. The geospatial distribution of total cancer cases attended at BMC Hospital for the study period of January 2019 to December 2021 revealed a heterogeneous distribution of different cancer types. Most patients attended at BMC came from Mwanza (47.5%), Mara (12.3%), Shinyanga (10.2%), Simiyu (8.3%), Kagera (7.5%) and Geita (6.1%), contributing above 200 cases each, as shown in **Figure 1** and **Table 1**.

**Table 1:** Social demographic characteristics of the registered cancer patients (Jan 2019 – Dec 2021).

Variables	N = 3697	%
Age category (years)		
0-17	426	11.5
18-34	321	8.7
35-51	1,001	27.0
52-68	1,274	34.5
69-98	675	18.3
Median age (IQR)	53(39-65)	
Sex		
Male	1,637	44.3
Female	2,060	55.7
Disease stage		
Stage 1	159	4.3
Stage 2	427	11.6
Stage 3	257	6.95
Stage 4	180	4.87
Disease not staged	2,674	72.3
HIV status		
Positive	70	1.90
Negative	47	1.27
Status unknown	3,575	96.8
Regional of residence		
Geita	225	6.1
Kagera	277	7.5
Kigoma	79	2.1
Mara	455	12.3
Mwanza	1,758	47.5
Shinyanga	376	10.2
Simiyu	307	8.3
Tabora	220	6.0
Residence status		
Urban	2007	54.3
Semi-urban	733	19.8
Rural	655	17.7
Unknown	302	8.2

IQR, interquartile range.

### 3.2 Geographical distribution of normalized common cancer cases per 100,000 population for January 2019 to December 2021

**Table 2** summarizes the distribution per 100,000 population over three years studied for different commonly diagnosed cancer cases. Mwanza, Shinyanga, and Mara regions had a higher case burden for cervix cancer among more than 10 per 100,000 population during the three-year study period. For prostate cancer, the Mwanza and Mara regions had a higher proportion of more than 5 diagnosed cases per 100,000 population in the last three years. For breast cancer, the Mwanza and Shinyanga regions had a higher proportion of diagnosed cases of more than 3.5 cases per 100,000 population between January 2019 to December 2021. Furthermore, Mwanza and Simiyu had a higher case burden of esophageal cancer cases per 100,000 population in the last three years compared to other regions.

**Table 2:** Overall regional distribution per 100,000 population of commonly diagnosed cancers (Jan 2019 – Dec 2021 (N = 3697)).

Variables	Region population*	Male Regional population*	Female Regional population*	Neoplasm of cervix uteri		Prostate cancer		Cancer of the breast		Neoplasm of the oesophagus	
Region				n	Pr**	n	Pr**	n	Pr**	n	Pr**
Geita	1,739,530	861,055	878,475	68	7.74	10	1.16	7	0.8	11	0.63
Kagera	2,458,023	1,205,683	1,252,340	46	3.67	42	3.48	30	2.4	34	1.38
Kigoma	2,127,930	1,028,994	1,098,936	8	0.73	6	0.58	13	1.18	4	0.19
Mara	1,743,830	840,020	903,810	117	12.95	43	5.12	28	3.1	20	1.15
Mwanza	2,772,509	1,360,381	1,412,128	308	21.81	237	17.4	193	13.67	143	5.16
Shinyanga	1,534,808	750,841	783,967	125	15.94	30	4.0	30	3.83	22	1.43
Simiyu	1,584,157	759,891	824,266	72	8.74	27	3.55	26	3.15	27	1.7

*Population as per 2012 census, **distribution per 100,000 population.

Majority (22%) of the participants were diagnosed with malignant neoplasm of the cervix cancer. About 11.2% of the participants were diagnosed with malignant neoplasm of the prostate. Also, 9.3% of the participants were diagnosed with malignant neoplasm of the breast, and about 7.6% of the participants were diagnosed with malignant neoplasm of the head and neck, **Table 3**.

**Table 3:** Cancer cases among study participants [Jan 2019 – Dec 2021 (N = 3697)].

Variables	N	%
Diagnosis		
*Malignant neoplasm of cervix Cancer*	813	22.0
*Malignant neoplasm of the prostate*	415	11.2
*Malignant neoplasm of breast*	342	9.3
*Malignant neoplasm of head and neck*	282	7.6
*Malignant neoplasm of oesophagus*	273	7.4
*Non-Hodgkin lymphomas*	200	5.4
*Malignant neoplasm of bladder*	174	4.7
*Malignant neoplasm of kidney*	121	3.3
*Malignant melanoma of the skin*	111	3.0
*Malignant neoplasm of the liver*	107	2.9
*Kaposi’s sarcoma*	106	2.9
*Leukemia*	72	2.0
*Malignant neoplasm of the lung*	48	1.3
*Malignant neoplasm of other connective and soft tissue*	47	1.3
*Malignant neoplasm of colon*	47	1.3
*Hodgkin lymphoma*	40	1.1
*Others*	499	13.5

### 3.3 Association between cancer cases and their determinants

*Cervix Cancer:* Participants from Shinyanga had a 70% higher proportion of diagnosed cervical cancer cases (aPR = 1.70, 95% CI = 1.51–1.79) as compared to other areas (See **Supplementary Material 1b**). Participants aged 52–68 had a 54% higher proportion of diagnosed cervix cancer (aPR = 1.54, 95% CI = 1.21–1.78), **Table 4**. Even after adjusting for other covariates, Shinyanga, Geita, and Tabora remained statistically significantly associated with the proportion of diagnosed cases of cervix cancer carcinomas.

**Table 4:** Association between neoplasm of cervix uteri and prostate cancer diagnosed cases and exposure variables.

Variables	*Malignant neoplasm of the cervix uteri*				*Malignant neoplasm of the prostate*			
	cPR (95%CI)	P-value	aPR (95%CI)	P-value	cPR (95%CI)	P-value	aPR (95%CI)	P-value
Age categories of the participants (years)								
0-17	1		1		1		1	
18-34	0.12(0.11, 1.28)	0.56	0.14(0.12, 1.43)	0.61	0.33(0.14, 1.56)	0.24	0.56(0.13,1.59)	0.25
35-51	0.92(0.78, 1.67)	0.13	0.91(0.73, 1.67)	0.11	0.45(0.04, 1.65)	0.57	0.45(0.04,1.68)	0.57
52-68	1.45(1.03, 1.78)	0.03	1.54(1.21, 1.78)	0.04	1.43(1.21, 1.84)	0.01	1.57(1.24,1.88)	0.01
69-98	1.65(0.13, 1.94)	0.24	1.54(0.13, 1.93)	0.12	1.65(1.03, 1.98)	0.04	1.75(1.12,1.99)	0.04
Region								
Geita	1.78(1.52,3.20)	0.03	1.82(1.59,4.29)	0.01	0.59(0.50,1.39)	0.19	0.59(0.51,1.38)	0.20
Kagera	0.53(0.24, 1.39)	0.13	0.59(0.16, 1.67)	0.28	1.66(1.13, 1.98)	0.03	1.75(1.13,1.94)	0.04
Kigoma	0.98(0.10,1.75)	0.49	0.76(0.50,1.51)	0.25	0.76(0.13,1.87)	0.20	0.44(0.13,1.56)	0.29
Mara	0.72(0.51,1.86)	0.12	0.87(0.57,1.96)	0.34	0.95(0.12,1.58)	0.14	0.53(0.12,1.56)	0.24
Mwanza	0.96(0.35, 2.80)	0.21	0.66(0.05, 2.80)	0.07	1.57(1.25, 2.50)	0.02	0.57(0.25,2.54)	0.08
Shinyanga	1.56(1.15,1.69)	0.01	1.49(1.15,1.87)	0.001	1.28(1.06,1.78)	0.01	1.58(1.06,1.78)	0.03
Simiyu	0.38(0.11,1.46)	0.10	0.35(0.03,1.37)	0.16	0.54(0.03,1.59)	0.65	0.51(0.15,1.56)	0.68
Tabora	1.29(1.10,2.12)	0.02	1.55(1.03,2.78)	0.03	0.84(0.63,2.15)	0.11	0.75(0.63,2.10)	0.21
HIV status								
Negative	1		1		1		1	
Positive	0.80(0.13,2.32)	0.12	0.54(0.11,1.63)	0.46	1.17(0.30,3.39)	0.81	0.65(0.12,1.59)	0.45
Residence status								
Unknown		1		1		1		1
Urban	1.12(0.61,3.78)	0.31	1.65(0.33,7.56)	0.72	1.28(0.35,4.19)	0.70	0.43(0.05,3.47)	0.44
Semi-urban	2.17(0.61,7.47)	0.37	1.84(0.31,7.18)	0.76	0.73(0.09, 3.72)	0.75	0.54(0.35,1.78)	0.59
Rural	0.87(0.16,2.67)	0.65	0.26(0.03,1.78)	0.16	1.14(0.36,3.78)	0.85	0.87(0.14,1.47)	0.77

*Prostate cancer:* In the crude analysis, Kahama, Kishapu, Muleba, Ngara, Nyangh’wale, and Sengerema districts were significantly associated with diagnosis of prostate cancer cases. Participants aged 69–98 years had a 75% higher proportion of diagnosis with prostate cancer (aPR=1.75, 95%CI:1.12–1.99), **Table 4**. Participants from Kahama had a 76% higher case burden of prostate cancer (aPR=1.76, 95%CI:1.31-1.98) as compared to other districts (See **Supplementary Material 1a**).

*Breast neoplasm*: In the preliminary analysis, age, Kahama, and Ngara district were significantly associated with an increased odds of breast neoplasm. Participants aged 35–51 years had 42% higher odds of having a neoplasm of the breast (aOR=1.42, 95%CI=1.06–2.09) (**Table 5**). Female participants had 44% higher odds of chance of being diagnosed with neoplasm of the breast (cOR=1.44, 95% CI = 1.38–1.61) as compared to male participants, **Table 5**. After controlling other variables, age, and sex, Kahama and Ngara districts remained statistically significantly associated with higher odds of neoplasm of the breast. Participants from Kahama district had 34% higher odds of chance of being diagnosed with neoplasm of the breast (aOR=1.34, 95%CI=1.12-4.68) as compared to their counterparts (See **Supplementary Material 2b**).

**Table 5:** Association between neoplasm of breast and head & neck diagnosed cases and exposure variables.

Variables	*Malignant neoplasm of the breast*				*Malignant neoplasm of head and neck*			
	cOR(95%CI)	P-value	aOR(95%CI)	P-value	cOR(95%CI)	P-value	aOR(95%CI)	P-value
Age categories of the participants (years)								
0-17	1				1		1	
18-34	1.50(0.91,2.45)	0.109	0.79(0.47,1.36)	0.40	0.29(0.18,0.47)	<0.001	0.34(0.19,0.59)	<0.001
35-51	2.10(1.79,3.68)	<0.001	1.42(1.06,2.09)	0.04	0.26(0.18,0.37)	<0.001	0.32(0.21,0.50)	<0.001
52-68	1.64(1.14,2.36)	0.01	1.05(0.71,1.56)	0.17	0.28(0.19,0.38)	<0.001	0.32(0.21,0.49)	<0.001
69-98	1.23(0.13,2.56)	0.43	1.25(0.11,2.59)	0.52	1.37(1.09,1.99)	<0.001	1.35(1.12,1.88)	<0.001
Sex								
Male	1		1		1		1	
Female	1.44(1.38,1.61)	<0.001	1.20(1.11,1.97)	0.01	1.59(1.46,1.75)	<0.001	1.43(1.29,1.82)	<0.001
Region of residency								
Geita	1.13(0.61,2.08)	0.68	0.60(0.36,1.20)	0.18	0.28(0.08, 2.40)	0.44	0.88(0.60,2.41)	0.49
Kagera	1.84(1.25,2.79)	0.02	1.53(1.11, 2.67)	0.04	0.49(0.10, 4.00)	0.56	0.43(0.20,4.08)	0.46
Kigoma	0.88(0.61,1.50)	0.19	1.60(1.29,3.20)	0.01	1.79(1.03,3.28)	0.02	1.78(1.04,3.09)	0.01
Mara	0.96(0.41,1.99)	0.97	0.75(0.02,2.12)	0.15	1.48(1.13,2.38)	0.03	1.75(1.13,2.39)	0.03
Mwanza	0.45(0.12, 3.49)	0.42	0.66(0.17,1.13)	0.09	0.82(0.51,2.45)	0.70	0.59(0.08,4.20)	0.59
Shinyanga	1.72(1.33,1.99)	0.02	0.74(0.13,2.30)	0.66	0.92(0.09,7.79)	0.23	1.16(0.51,2.46)	0.71
Simiyu	0.80(0.17,3.43)	0.71	0.65(0.11,0.89)	0.12	0.67(0.34,3.86)	0.79	0.90(0.09,1.79)	0.23
Tabora	0.70(0.65,4.43)	0.26	0.60(0.22,3.20)	0.17	0.48(0.10, 4.05)	0.49	0.77(0.36,3.87)	0.70
HIV status								
Negative	1		1		1		1	
Positive	1.30(0.19,2.96)	0.67	0.68(0.15,1.87)	0.21	1.10(0.08,1.36)	0.18	0.69(0.14,1.80)	0.43
Residence status								
Unknown		1		1		1		1
Urban	0.67(0.31,2.67)	0.65	0.95(0.33,2.71)	0.92	0.78(0.38,2.67)	0.66	0.94(0.32,2.76)	0.96
Semi-urban	0.75(0.23,2.56)	0.53	0.77(0.26,2.26)	0.63	0.75(0.21,2.57)	0.54	0.71(0.26,2.45)	0.53
Rural	1.54(1.07,2.86)	0.04	1.34(1.01,2.15)	0.04	1.37(1.07,2.83)	0.04	1.31(1.0,2.15)	0.04

*Head and neck neoplasm*: In the preliminary analysis, the Kasulu and Musoma districts were significantly associated with the risk of head and neck neoplasm. Female participants had 59% higher odds of being diagnosed with neoplasm of head and neck (cOR=1.59, 95% CI = 1.46-1.75) than male participants, **Table 5**. After adjusting for other variables such as age, gender, and district, participants aged 69–98 years had a 35% higher odds of being diagnosed with head and neck neoplasm (aOR=1.35, 95% CI = 1.12-1.88) than their reference group aged 0–17 years; female participants had a 43% higher odds of being diagnosed with head and neck neoplasm (aOR = 1.43, 95% CI = 1.29-1.82) than their counterparts, **Table 5**.

*Neoplasm of the oesophagus*: In the crude analysis, Bariadi, and Geita district were significantly associated with higher odds of neoplasm of the oesophagus. Participants aged 69–98 years had 42% higher odds of being diagnosed with neoplasm of the oesophagus (cOR = 1.42, 95% CI = 1.10–2.76) as compared to their reference group aged 0–17 years (**Table 6**). Participants from Bariadi district had 34% higher odds of being diagnosed with neoplasm of the oesophagus (cOR = 1.34, 95% CI = 1.11-2.53) as compared to reference group. Participants from Geita district had 43% higher odds of being diagnosed with neoplasm of oesophagus (cOR = 1.43, 95%CI = 1.16-1.97) as compared to their counterparts (See **Supplementary Material 3a**). Participants aged 69–98 years had 69% higher odds of being diagnosed with neoplasm of the oesophagus (aOR = 1.69, 95% CI = 1.13-2.67) than their reference group aged 0–17 years, **Table 6**. Participants from Bariadi district had 35% higher odds of being diagnosed with neoplasm of the oesophagus (aOR = 1.35, 95% CI = 1.22-1.87) than male participants, (See **Supplementary Material 3a**).

**Table 6:** Association between neoplasm of the esophagus and non-Hodgkin lymphomas and the exposure variables.

Variables	*Malignant neoplasm of the oesophagus*				*Non-Hodgkin lymphomas*			
	cOR(95%CI)	P-value	aOR(95%CI)	P-value	cOR(95%CI)	P-value	aOR(95%CI)	P-value
Age categories of the participants (years)								
0-17	1				1		1	
18-34	0.93(0.81,1.45)	0.14	0.76(0.33,1.45)	0.30	0.45(0.16,0.78)	<0.001	0.35(0.19,0.69)	<0.001
35-51	0.57(0.29,3.43)	0.41	0.67(0.34,2.23)	0.31	0.28(0.13,0.37)	0.02	0.67(0.21,0.90)	<0.001
52-68	1.65(1.31,2.14)	0.02	1.67(1.41,1.98)	0.03	0.67(0.19,0.69)	0.01	0.34(0.21,0.78)	0.01
69-98	1.42(1.10,2.76)	0.01	1.69(1.13,2.67)	0.02	0.56(0.09,0.89)	0.01	0.16(0.09,0.87)	0.03
Sex								
Male	1		1		1		1	
Female	0.94(0.48,1.67)	0.63	0.89(0.34,1.94)	0.57	0.67(0.48,0.79)	<0.001	0.78(0.49,0.82)	<0.001
Region								
Geita	1.44(1.11,2.53)	0.02	1.55 (1.22,1.89)	0.01	1.25(0.68, 2.67)	0.48	1.54(0.61,2.48)	0.43
Kagera	0.67(0.20,2.79)	0.67	0.56(0.14, 2.60)	0.41	0.68(0.40, 3.56)	0.51	0.48(0.16,3.06)	0.35
Kigoma	0.85(0.62,1.59)	0.16	0.30(0.24,1.27)	0.27	0.81(0.03,1.96)	0.28	0.57(0.12,3.09)	0.61
Mara	0.67(0.49,1.97)	0.80	0.75(0.12,2.14)	0.19	2.20(1.11,4.65)	0.04	1.47(1.23,1.91)	0.04
Mwanza	0.56(0.12, 2.49)	0.31	0.75(0.25,1.13)	0.37	1.19(0.50,2.43)	0.73	0.68(0.04,4.76)	0.53
Shinyanga	0.68(0.39,1.74)	0.28	0.68(0.11,4.30)	0.87	1.95(0.07,7.79)	0.33	1.68(1.11,2.45)	0.01
Simiyu	1.78(1.21,3.78)	0.04	1.69(1.14,5.84)	0.02	1.76(1.08,3.80)	0.03	2.21(1.06,7.99)	0.03
Tabora	0.69(0.50,2.47)	0.47	0.73(0.25,4.24)	0.12	1.46(0.54, 4.01)	0.43	1.18(0.24,3.81)	0.76
HIV status								
Negative	1		1		1		1	
Positive	1.21(0.10,2.07)	0.33	0.59(0.14,1.75)	0.69	1.18(0.22,1.72)	0.19	0.68(0.13,1.99)	0.43
Residence status								
Unknown		1		1		1		1
Urban	0.34(0.31,2.62)	0.64	0.95(0.32,2.71)	0.94	0.43(0.34,2.67)	0.76	0.96(0.22,2.76)	0.95
Semi-urban	0.72(0.23,2.54)	0.52	0.77(0.26,2.23)	0.62	0.65(0.21,2.59)	0.59	0.73(0.16,2.45)	0.57
Rural	1.51(1.07,2.87)	0.01	1.34(1.01,2.15)	0.04	1.48(1.17,2.83)	0.03	1.34(1.12,2.15)	0.04

1. Bray, F., et al., Global cancer statistics 2022: GLOBOCAN estimates of incidence and mortality worldwide for 36 cancers in 185 countries. CA: a cancer journal for clinicians, 2024. **74**(3): p. 229-263.

2. Kimario, O.M., et al., Pattern of Head and Neck Malignancies and Associated Factors for Late Diagnosis at Bugando Medical Centre. Clinical Case Reports and Clinical Study, 2024. **11**(1).

*Non-Hodgkin’s lymphomas*: In the crude analysis, the Busega and Musoma districts were significantly associated with the odds of being diagnosed with neoplasm of non-Hodgkin’s lymphomas. Participants from the Busega district had 18% higher odds of being diagnosed with neoplasm of non-Hodgkin’s lymphomas (cOR = 1.18, 95% CI = 1.07-3.87) as compared to their reference group (See **Supplementary Material 3a**). Participants from Musoma had two times higher odds of being diagnosed with neoplasm of non-Hodgkin’s lymphomas (cOR = 2.27, 95% CI = 1.16-4.06) as compared to control participants (See **Supplementary Material 3b**). After adjusting for other variables, the Busega and Musoma districts remained statistically significantly associated with higher odds of being diagnosed with neoplasm of non-Hodgkin’s lymphomas. Participants from the Busega district had two times higher odds of being diagnosed with neoplasm of non-Hodgkin’s lymphomas (aOR = 2.30, 95% CI = 1.09–7.77) (See **Supplementary Material 3a**). Participants from Musoma had two times higher odds of being diagnosed with neoplasm of non-Hodgkin’s lymphomas (aOR = 2.21, 95% CI = 1.17–4.06) as compared to their controls (See **Supplementary Material 3b**).

## 4 Discussion

### 4.1 Overview of Mwanza cancer registry

This study identified substantial data incompleteness, which limited the ability to perform robust inferential analyses. For example, over 68% of patients’ records had no occupation status recorded. Also, more than 96% of the records had no HIV status recorded. Furthermore, 99% of the patients’ health status was recorded as alive, contrary to a ten-year retrospective hospital-based study published in Tanzania on cancer mortality patterns which indicated increasing mortality rates (25). More than 72% of the patients’ cancer staging was not indicated similar to the observation back in 2016 where over 77% of the diagnoses were not staged, indicating a challenge in improving data quality within the MCR management (16). Such challenges have been reported at a similar registry in Kilimanjaro Region in Northern Tanzania where a lack of understanding of the importance of improving the quality and completeness of information among involved healthcare workers was raised (26). The WHO has cautioned against the absence and/or incompleteness of cancer registries in LMICs which could potentially underestimate the actual disease burden, and as a result, could affect patient management due to incomplete data, hence poor planning (27, 28).

### 4.2 Demographic characteristics of study participants

The median age of participants was 53 (39–65), older than the prior analysis from 2016, which was 46 years old (16) consistently with a South Africa study on cancer mortality and trend which observed that more cancer cases were from a similar age group (29). Cancer is a chronic disease that takes a long time to manifest symptoms and may take more than 10 years to develop (30). Given the typically long latency period in cancer development, the finding that nearly 20% of registered cases occurred among individuals under 30 years suggests an early age of disease onset, indicating that carcinogenic exposures may have been initiated in early life. The higher number of cases could be due to improvement in pediatric and adolescent cancer awareness, and environmental exposures around Lake Zone. This is consistent with the report on cancer statistics published in 2020, which observed an increase and predicted more increases in cancer cases among adolescents and young adults (31). This was reported to be caused by several factors including improvement in awareness and case finding, Ultraviolet light exposure in the young generation, early onset of disease regardless of heredity, and excessive body weight among the young generation.

It was observed that the number of female cancer patients was higher as compared to male patients (55.7% vs 44.3%), consistent with previous studies (50.7% to 60% in 2016 and 2020, respectively) (16, 25). However, this is different from the study done in Turkey, where the proportion of cancer cases in males exceeded those found in females (32). This could be due to a difference in culture, norms, and lifestyle as well as healthcare-seeking behavior differences between Turkey and Tanzania. On the other hand, most of the health promotion campaigns have been centered on females as compared to men, influencing their health-seeking behavior (33). Regarding the residence status, most of the participants were from urban areas (54% vs 19%). This could be due to an increase in the migration of people from rural to urban residences and a change in urban lifestyle that increases exposure to cancer risk factors.

### 4.3 Cancer patterns among study participants

In the current study, the leading common cancers are the cancer of cervix cancer, cancer of prostate, cancer of breast, cancers of head and neck, and cancer of the esophagus. Previously, a review of the MCR revealed almost a similar pattern where cervical cancer and breast cancer were the leading cancers. The previous MCR review also reported high HIV/AIDS related carcinomas which is not the case in the current study, this could be because, around the year 2016, BMC Oncology Department had a special HIV-related cancer campaign (16). The findings are consistent with the GLOBOCAN report on Mapping Cancer in Africa, which shows that Cervical Cancer is the most common cancer in this region (34).

These results are consistent with a 2019 10-year cancer trend study conducted in Tanzania (17). This study observed a relatively high proportion of prostate cancer (9.1%), ranking second after uterine and cervical cancers among patients at BMC, consistent with reports from other African settings where prostate cancer burden is high (34). However, this is contrary to the Tanzania Ministry of Health report (2018), which ranked prostate cancer seventh (35). Early diagnosis and timely treatment are critical for effective cancer management. In this study, only 16% of patients presented with early-stage disease (stage I–II), suggesting generally delayed presentation. The observed proportion of early-stage diagnoses may reflect ongoing community-based cancer awareness efforts aimed at improving health-seeking behavior in the Lake Zone. Previous studies published at BMC in 2012 and 2015 on breast cancer and head & neck cancers observed that more than 95% of patients had an advanced stage at the time of diagnosis (36, 37). Late presentation in healthcare is the major factor of poor prognosis of cancer in LMICs where patients present at the hospital with advanced stages of the disease, and this is due to several factors in the pathway to diagnosis and treatment (15, 38, 39).

### 4.4 Geospatial distribution of cancer cases among study participants and associated factors

In the current study, Mwanza, Mara, and Shinyanga had more cases than other regions by 47.5%, 12.5%, and 10.5%, respectively. Normalization of common occurring cancers per 100,000 population for the studied period these regions continue to have distribution as seen in **Table 4** above. In the last six years, more than 54% of cases came from the Mwanza region (16). This means there is a slight increase in the number of patients from other regions in the Lake Zone. The uneven distribution of cancer cases across regions may reflect geographic differences in healthcare utilization, diagnostic ascertainment and exposure to cancer risk factors.

The leading diagnosis was cancer (malignant) of the uterine cervix. This is consistent with the disease’s natural history, in which the disease takes more than three decades to develop after exposure to the human papillomavirus (HPV) (40). Also, it is consistent with the study done in Tanzania, where the leading age group was reported to be 50-plus years old (41). Infection by HPV is the most common and important risk factor for cervical cancer (40, 42). The Lake Zone is known for its high case burden of schistosomiasis, especially Schistosoma *hematobium* infection, which increases the risk of HIV and HPV (43, 44). Recent research has found that areas endemic to female genital schistosomiasis have higher registered cases of cervical and uterine cancer in infected patients than the general population, and the Lake Zone is no exception (45–47). Furthermore, patients from Shinyanga, Geita, and Tabora were significantly associated with higher proportions of uterine and cervical cancers. However, participants from Shinyanga had a 25% higher case composition of cervix uteri as compared to other districts. On normalization to 100,000 population over a study period Kahama, Shinyanga, Misungwi, Musoma, Nyamagana, Sengerema and Tabora districts had the higher case burden of more than 17 diagnosed cervical cancer cases forming hotspots for this type of cancer among individuals attended at BMC. Other risk factors for this cancer include early and/or childhood sexual intercourse, as well as multiple sexual partners and early child marriages (48).

Prostate cancer in this study shows a high proportion (11.2%) being second to uterine and cervical cancers compared with other types among the patients attended at BMC. This is explained by the fact that schistosomiasis is endemic in the Lake Zone, and published studies have established a link between schistosomiasis and the risk of prostate cancer (49, 50). A high number of prostate cancer cases were associated with Kahama, Kishapu, Muleba, Ngara, Nyangh’wale, and Sengerema, which are areas in the Lake Zone where schistosomiasis is reported to be common (51). When normalized per 100,000 population over the study period Kahama, Nyamagana, Sengerema and Urambo districts had higher proportion of diagnosed cases of more than 5 Prostate cancer patients, making the disease hotspots.

Neoplasm of the Breast is among the top three cancers in the current study, with a proportion of 9.3% among the studied patient records. Participants aged 35 to 51 had higher odds of developing this type of cancer. This is consistent with the 9-year retrospective study done at BMC Mwanza published in 2011 where more than 50% of patients were below the age of 46 years (52). Previously, more than 60% of the patients with neoplasm of the breast at BMC Hospital were reported to be pre-menopausal stage (36). This trend could be due to a change in lifestyle among Tanzanians. Also, this study observed that living in Kahama and Ngara Districts is associated with greater odds of developing breast cancer. However, normalizing the cases per 100,000 population over three consecutive years Kahama, Bariadi, Geita, Magu and Musoma Districts had a higher case burden of more than 4 breast cancer diagnosed cases. In addition, these hotspot areas are mining/nearby mining zones or there is high movement of people between these areas and the areas with large mining activities and the use of heavy metals is not uncommon (19). Studies done elsewhere in the world reveal recent evidence of the risk of heavy metals to breast cancer. However, this needs more studies, especially in our settings (53).

**Figure 2:**
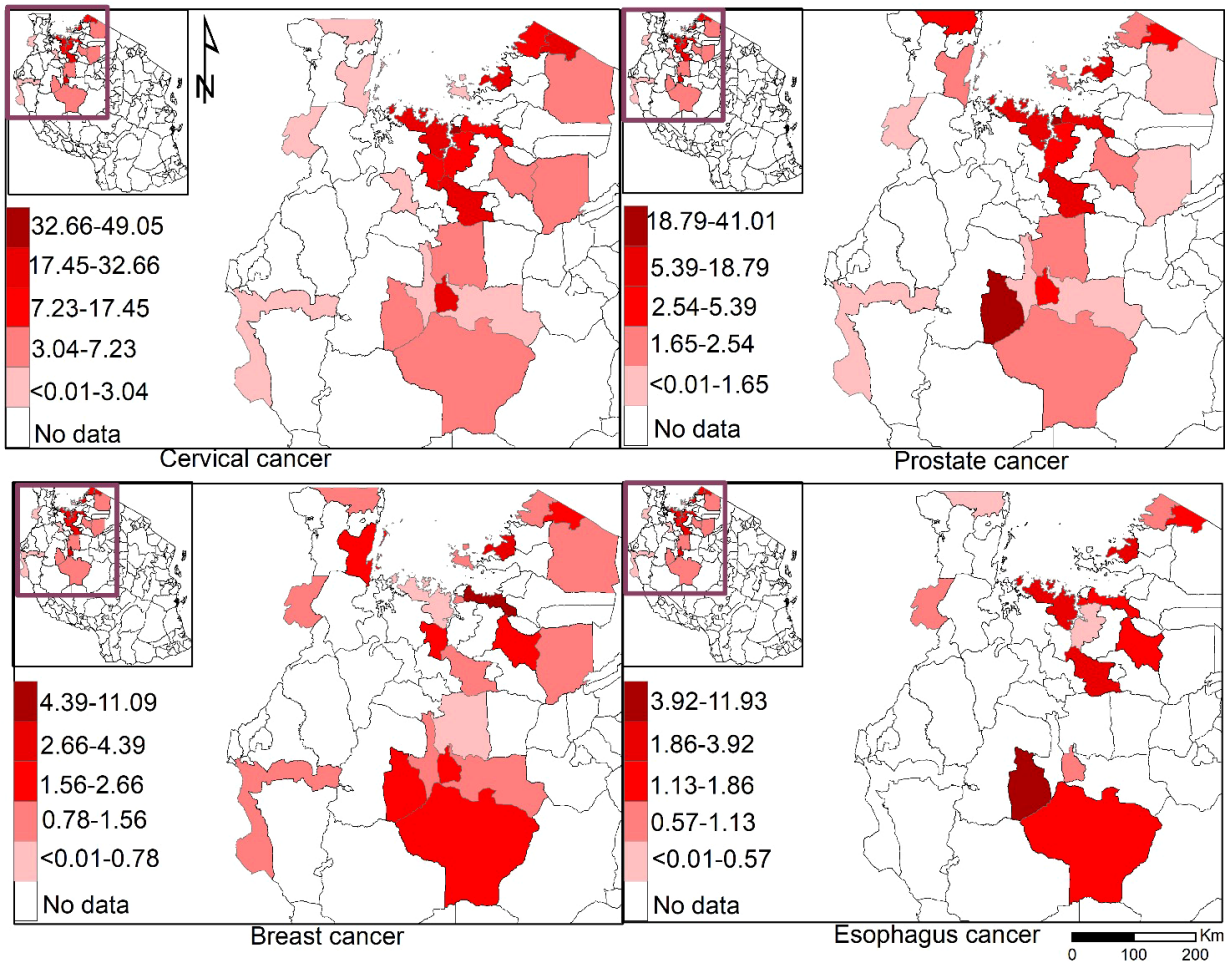
Distribution of cancer cases per 100,000 geographical area population in Lake Zone districts [Jan 2019 – Dec 2021 (N = 3697)]. Adapted from (24).

In the current study, malignant neoplasm of the esophagus is among the cancers with a high proportion of cases, with a proportion of 7.4%. Participants aged 69 to 98 had higher odds of developing this type of cancer. This is consistent with the study done at Muhimbili and Ocean Road Cancer Institute in the Eastern part of Tanzania published in 2022 showing an increase in esophageal cancer and the majority of the participants were above the age of 45 years (54). Also, this study observed that living in Kasulu and Musoma Districts is associated with greater odds of being diagnosed with esophageal cancer. However, the cancer composition per 100,000 population revealed that Bunda, Kahama, Musoma, Sengerema and Urambo Districts had higher registered cases of esophageal cancer diagnosed (**Figure 2**). Studies have reported smoking, exposure to raw nuts, and low social status as associated factors with this type of cancer (55). In the current study, non-Hodgkin’s lymphomas also contributed to a large number of cases, with a proportion of 5.4%. This was observed to be a common neoplasm among pediatric cases at BMC. This is consistent with the studies done here at BMC in 2017 and 2021; non-Hodgkin’s lymphoma was found to be among the top three common malignancies among pediatric patients (56). This study also observed that most cases come from Busega and Musoma districts.

Even though not all cancers are due to environmental exposure. Research has established that environmental factors surrounding human beings (external carcinogens) together with genetic factors are responsible for over 80% of cancers (57, 58). For instance, in the current study, among the recorded kidney cancers (malignant neoplasm of the kidney), 73.6% were reported in participants aged less than 20 years. This concurs with an international population-based study published in 2020 where the proportion of diagnosed renal cancer cases in children was reported to increase (59). Although kidney cancer is linked to genetic risk factors, the environmental contribution is not to be ignored. The current study indicates that about 40% of all renal carcinomas are coming from only four districts in the Lake Zone, namely Bariadi in the Simiyu region, Sengerema in Mwanza region, as well as Ilemela and Nyamagana in Mwanza City, all of which are of mixed economic activities involving fishing, mining, and farming, like many areas in the region.

Geospatial patterns revealed heterogeneity in cancer distribution across districts, with identifiable high- and low-burden areas. This underscores the need to prioritize hotspot regions for further investigation to better understand underlying risk factors and causal pathways, and to inform targeted intervention strategies. Strengthening the Mwanza Cancer Registry (MCR) to improve data completeness and quality is essential. Given the observed geographic variation, public health efforts such as awareness campaigns, promotion of health-seeking behavior, and early screening should be strategically focused in high-burden areas to facilitate early diagnosis and improve prognosis.

A key strength of this study is its large sample size (>3,000 participants), which provides robust insights into cancer patterns, trends, and potential geographic hotspots in the Lake Zone. Although it does not estimate true case burden, it offers valuable information on patients’ origins, cancer types, and possible risk factor distribution. The geospatial analysis enhances understanding of where and among whom cancer burden is concentrated, thereby informing targeted research, interventions, and health planning across the Lake Zone, including Tabora and Kigoma regions. Overall, the study helps fill an important public health knowledge gap on cancer distribution and associated factors in this setting.

The study may suffer from several limitations. Firstly, the analysis relied on routinely collected registry and hospital data, which contained substantial missing information such as occupation, HIV status, treatment information and patient outcomes. This incompleteness may have introduced information bias and reduced the ability to comprehensively assess potential demographic, clinical, environmental and behavioral determinants of cancer occurrences. Secondly, the absence of key covariates may have resulted in residual confounding within the regression models, potentially affecting the accuracy of the reported associations. Some individuals may not have been diagnosed due to inability to afford diagnostic tests, while others may have failed to attend the clinic due to financial, socio-economic, or socio-cultural barriers, potentially leading to underestimation of the true disease burden in the Lake Zone. Additionally, patients with advanced disease may be less likely to present to health facilities in this setting (44). Late-stage patients may have been less likely to seek care, introducing potential selection bias. Finally, residence data, based on self-report, may have been misclassified, leading to possible geographic misrepresentation and ecological fallacy. However, 75% of records were validated by phone. Additionally, lack of survival data may have led to overestimation of case burden, although cancer’s chronic nature suggests prolonged survival in many cases. Despite these limitations, the large sample size and use of cancer registry data provide valuable insights into cancer patterns and geographic variations in northwestern Tanzania.

## 5 Conclusion

Diagnosed cancer cases in the Lake Zone are unevenly distributed, with some areas presenting more diagnosed patients as compared to others. Distinct geographical hotspots were identified for different cancer types in different districts in the Lake Zone that can be used for cancer programs in the region. Cervix cancer and prostate cancer were the most common among all patients diagnosed at BMC in the last three years. The burden of cervical cancer is high in the Lake Zone.

## Data Availability

The raw data supporting the conclusions of this article will be made available by the authors, without undue reservation.
