# Supplementary material for: Geospatial distribution and associated factors of cancer cases registered at a tertiary hospital in Northwestern Tanzania. A retrospective study
Source: Front Oncol. 2026 Jul 22;16:1859286. doi: 10.3389/fonc.2026.1859286 (PMC13437503; doi:10.3389/fonc.2026.1859286)
Supplement: Supplementary file 1 [file DataSheet1.pdf]

Supplementary Materials

Supplementary material 1a: Crude and Adjusted Prevalence ratio between neoplasm of cervix uteri and prostate diagnosed cases and districts in Lake Zone

	**Malignant neoplasm of the cervix uteri**				**Malignant neoplasm of prostate**			
**Variables**	**cPR(95%CI)**	**P-value**	**aPR (95%CI)**	**P-value**	**cPR(95%CI)**	**P-value**	**aPR(95%CI)**	**P-value**
**District**								
Bariadi	0.86(0.59,1.26)	0.43	0.83(0.59,1.27)	0.41	0.56(0.55,1.34)	0.21	0.57(0.55,1.38)	0.22
Biharamulo	0.59(0.26, 1.35)	0.22	0.58(0.16, 1.45)	0.20	0.65(0.13, 1.65)	0.13	0.67(0.13,1.64)	0.19
Buhigwe	0.72(0.15,1.43)	0.25	0.75(0.55,1.45)	0.25	0.46(0.13,1.54)	0.23	0.44(0.13,1.53)	0.21
Bukoba	0.80(0.51,1.26)	0.34	0.80(0.51,1.26)	0.31	1.35(1.12,1.58)	0.04	1.53(1.12,1.56)	0.04
Bukombe	1.66(0.95, 2.89)	0.07	1.66(0.95, 2.89)	0.09	0.57(0.25, 2.54)	0.08	0.57(0.25,2.54)	0.08
Bunda	1.09(0.75,1.62)	0.64	1.09(0.75,1.62)	0.66	1.23(0.56,1.78)	0.41	1.22(0.56,1.78)	0.45
Busega	0.35(0.09,1.32)	0.12	0.35(0.09,1.32)	0.16	0.54(0.05,1.56)	0.65	0.51(0.05,1.56)	0.67
Butiama	1.21(0.69,2.12)	0.25	1.21(0.63,2.12)	0.23	1.34(0.63,2.15)	0.11	1.35(0.63,2.15)	0.21
Chato	1.17(0.97,2.55)	0.07	1.21(0.97,2.55)	0.07	1.45(0.93,2.61)	0.09	1.46(0.93,2.67)	0.06
Geita	1.40(1.02,1.48)	0.03	1.56(1.33,1.65)	0.03	1.46(0.04,1.64)	0.53	1.47(0.03,1.64)	0.57
Igunga	1.52(0.86,2.69)	0.15	1.51(0.86,2.69)	0.17	1.23(0.45,2.76)	0.54	1.24(0.45,2.76)	0.58
Ilemela	0.71(0.57,0.87)	0.001	0.74(0.57,0.87)	0.001	0.56(0.43,1.87)	0.24	0.58(0.43,1.87)	0.36
Itilima	1.21(0.42,1.92)	0.21	1.45(0.41,1.95)	0.34	1.53(0.45,1.64)	0.43	1.57(0.45,1.69)	0.44
Kahama	1.84(1.42,1.92)	0.01	1.85(1.42,1.93)	0.01	1.75(1.32,1.97)	0.01	1.76(1.31,1.98)	0.01
Kakonko	1.24(0.34,2.81)	0.32	1.24(0.34,2.81)	0.32	1.56(0.22,2.76)	0.45	1.54(0.22,2.76)	0.45
Kaliua	0.92(0.33,1.56)	0.57	0.95(0.33,1.56)	0.51	0.44(0.11,1.67)	0.21	0.44(0.11,1.67)	0.28
Karagwe	0.81(0.52,1.51)	0.41	0.83(0.52,1.51)	0.40	0.56(0.04,1.87)	0.34	0.56(0.04,1.87)	0.34
Kasulu	0.62(0.31,0.98)	0.01	0.62(0.31,0.98)	0.01	0.36(0.23,1.97)	0.51	0.37(0.23,1.97)	0.52
Kibondo	0.75(0.52,0.98)	0.02	0.75(0.52,0.98)	0.03	0.43(0.61,1.99)	0.44	0.43(0.61,1.99)	0.48
Kigoma	0.98(0.22,1.82)	0.15	0.95(0.22,1.82)	0.41	0.56(0.21,1.67)	0.56	0.55(0.21,1.67)	0.56
Kishapu	1.71(0.49,1.82)	0.25	1.71(0.49,1.82)	0.25	1.32(1.23,1.97)	0.03	1.65(1.24,1.96)	0.03
Kwimba	0.98(0.45,1.56)	0.76	0.98(0.45,1.56)	0.76	0.86(0.34,1.59)	0.67	0.96(0.34,1.63)	0.65
Kyerwa	0.98(0.43,0.99)	0.01	0.92(0.43,0.99)	0.02	0.67(0.54,1.89)	0.45	0.68(0.54,1.89)	0.45

Supplementary material 1b. Crude and Adjusted Prevalence ratio between neoplasm of cervix uteri and prostate diagnosed cases and district

	**Malignant neoplasm of the cervix uteri**				**Malignant neoplasm of prostate**			
**Variables**	**cPR(95%CI)**	**P-value**	**aPR(95%CI)**	**P-value**	**cPR(95%CI)**	**P-value**	**aPR(95%CI)**	**P-value**
Magu	0.75(0.51,1.88)	0.67	0.75(0.51,1.88)	0.67	0.46(0.53,1.45)	0.54	0.46(0.53,1.45)	0.54
Maswa	0.91(0.57,0.87)	0.001	0.91(0.57,0.87)	0.01	0.45(0.34,0.89)	0.02	0.45(0.34,0.89)	0.02
Mbogwe	1.75(0.12,1.81)	0.19	1.76(0.12,1.81)	0.19	1.43(0.11,1.56)	0.34	1.43(0.14,1.56)	0.34
Meatu	0.76(0.56,1.89)	0.43	0.76(0.56,1.89)	0.44	0.66(0.43,1.65)	0.32	1.66(1.43,1.69)	0.03
Missenyi	0.58(0.55,0.85)	0.01	0.52(0.55,0.85)	0.01	0.43(0.12,1.89)	0.17	0.49(0.12,1.89)	0.19
Misungwi	0.59(0.57,0.87)	0.02	0.59(0.57,0.87)	0.04	0.56(0.43,1.88)	0.34	0.58(0.43,1.89)	0.34
Muleba	0.74(0.57,1.82)	0.23	0.76(0.57,1.82)	0.23	1.45(1.34,1.56)	0.03	1.47(1.34,1.56)	0.03
Musoma	0.99(0.52,1.83)	0.45	0.97(0.52,1.83)	0.45	0.65(0.54,1.65)	0.34	0.65(0.54,1.65)	0.34
Ngara	0.82(0.57,1.86)	0.16	0.82(0.57,1.86)	0.18	1.67(1.54,1.86)	0.02	1.66(1.53,1.89)	0.02
Nzega	0.11(0.07,1.45)	0.67	0.15(0.07,1.45)	0.68	0.34(0.31,1.65)	0.56	0.34(0.31,1.65)	0.59
Nyamagana	1.57(1.51,1.87)	0.02	1.56(1.51,1.87)	0.02	1.46(1.32,1.88)	0.04	1.48(1.32,1.88)	0.04
Nyang'hwale	0.65(0.57,0.87)	0.001	0.65(0.57,0.87)	0.001	0.67(0.55,0.69)	0.03	0.62(0.55,0.69)	0.03
Rorya	0.71(0.51,1.87)	0.11	0.71(0.51,1.87)	0.11	0.45(0.32,1.98)	0.32	0.45(0.32,1.98)	0.37
Sengerema	0.56(0.37,1.81)	0.35	0.56(0.37,1.81)	0.35	1.68(1.54,1.78)	0.01	1.65(1.54,1.78)	0.01
Serengeti	0.89(0.02,1.72)	0.67	0.89(0.02,1.72)	0.67	0.67(0.12,1.56)	0.34	0.67(0.12,1.56)	0.34
Shinyanga	1.25(1.14,1.76)	0.01	1.70(1.51,1.79)	0.02	1.34(1.31,1.78)	0.04	1.36(1.31,1.78)	0.04
Sikonge	0.75(0.51,1.88)	0.67	0.75(0.51,1.88)	0.69	0.37(0.34,1.97)	0.62	0.37(0.34,1.97)	0.68
Tabora mc	1.35(1.17,1.82)	0.01	1.38(1.14,1.86)	0.01	1.23(0.04,1.56)	0.39	1.24(0.04,1.56)	0.36
Tarime	0.65(0.51,1.45)	0.54	0.64(0.51,1.45)	0.53	0.45(0.23,1.57)	0.33	0.42(0.23,1.57)	0.33
Ujiji MC	0.76(0.23,1.85)	0.57	0.75(0.23,1.85)	0.58	0.43(0.12,1.76)	0.69	0.43(0.12,1.76)	0.65
Ukelewe	0.85(0.45,1.76)	0.43	0.85(0.45,1.76)	0.29	0.56(0.41,1.63)	0.12	0.55(0.41,1.63)	0.17
Urambo	0.11(0.02,1.80)	0.32	0.15(0.02,1.80)	0.33	0.15(0.11,1.87)	0.45	0.18(0.11,1.87)	0.49
Uvinza	0.65(0.57,0.87)	0.01	0.67(0.57,0.87)	0.01	0.69(0.54,0.97)	0.01	0.66(0.54,0.89)	0.01
Uyui	0.75(0.42,1.86)	0.46	0.76(0.42,1.86)	0.43	0.44(0.39,1.87)	0.31	0.32(0.39,1.86)	0.34

Supplementary material 2a. Crude and Adjusted Odds ratio between neoplasm of breast and head and neck diagnosed cases and districts in the Lake Zone

	**Malignant neoplasm of the breast**				**Malignant neoplasm of head and neck**			
**Variables**	**cOR(95%CI)**	**P-value**	**aOR(95%CI)**	**P-value**	**cOR(95%CI)**	**P-value**	**aOR(95%CI)**	**P-value**
Magu	0.61(0.24,1.51)	0.28	0.48(0.18,1.29)	0.14	0.75(0.30,1.87)	0.54	0.75(0.30,1.87)	0.53
Maswa	0.98(0.35,2.76)	0.42	0.58(0.13,2.56)	0.08	0.57(0.14,2.38)	0.44	0.59(0.14,2.38)	0.47
Mbogwe	0.73(0.17,3.06)	0.66	1.15(0.32,3.74)	0.81	2.23(0.73,8.41)	0.19	2.26(0.73,8.41)	0.14
Meatu	0.75(0.09,5.78)	0.13	0.42(0.07, 2.52)	0.34	0.43(0.06,3.18)	0.41	0.47(0.06,3.18)	0.40
Missenyi	0.75(0.15,3.04)	0.66	1.23(0.10,14.4)	0.17	0.93(0.12,7.14)	0.95	0.94(0.12,7.14)	0.89
Misungwi	1.45(0.56,3.73)	0.24	1.58(0.36,6.96)	0.54	1.20(0.55,2.63)	0.65	1.23(0.55,2.63)	0.67
Muleba	0.12(0.17,3.06)	0.67	0.28(0.09,0.81)	0.02	1.01(0.31,3.29)	0.99	1.21(0.31,3.29)	0.98
Musoma	0.44(0.16,1.22)	0.11	0.42(0.17,1.25)	0.14	2.26(1.28,4.06)	0.01	2.24(1.27,4.06)	0.01
Ngara	1.64(1.37,7.35)	0.02	1.05(1.01, 5.73)	0.04	0.54(0.03,3.34)	0.47	0.57(0.03,3.34)	0.48
Nzega	0.41(0.10,1.71)	0.22	0.29(0.07,1.28)	0.12	1.70(0.72,4.04)	0.23	1.73(0.72,4.04)	0.26
Nyamagana	1.36(1.03,1.79)	0.03	0.84(0.58,1.21)	0.34	1.10(0.79,1.52)	0.55	1.14(0.79,1.52)	0.59
Nyang'hwale	1.65(0.31,7.35)	0.51	1.43(0.51,7.34)	0.53	0.83(0.07,0.99)	0.01	0.85(0.07,0.99)	0.01
Rorya	0.33(0.08,1.38)	0.12	0.20(0.05,0.87)	0.03	0.86(0.31,2.39)	0.78	0.86(0.31,2.39)	0.74
Sengerema	1.18(0.84,1.67)	0.43	1.04(0.27, 3.97)	0.95	0.78(0.51,1.20)	0.27	0.75(0.51,1.20)	0.27
Serengeti	1.13(0.34,3.76)	0.84	1.34(0.22, 3.56)	0.58	0.43(0.06, 3.18)	0.41	0.48(0.26,3.18)	0.49
Shinyanga	0.55(0.28,1.09)	0.09	0.37(0.17,0.77)	0.01	1.04(0.58,1.85)	0.90	1.04(0.58,1.85)	0.98
Sikonge	1.09(0.43,2.77)	0.16	1.43(0.21,2.65)	0.17	1.62(0.37,7.12)	0.52	1.57(1.33,7.12)	0.02
Tabora MC	0.59(0.21,1.63)	0.32	0.47(0.18,1.26)	0.13	1.05(0.38,2.95)	0.92	1.25(0.38,2.95)	0.96
Tarime	1.09(0.43,2.76)	0.35	0.32(0.11,1.94)	0.04	0.93(0.37,2.33)	0.88	0.94(0.37,2.33)	0.86
Ujiji MC	4.40(1.35,14.3)	0.01	4.48(1.08,18.5)	0.04	1.01(0.13,7.79)	0.99	1.01(0.13,7.79)	0.99
Ukelewe	0.91(0.32,2.55)	0.86	0.59(0.14,2.61)	0.49	2.15(0.95,4.84)	0.06	2.65(0.95,4.84)	0.07
Urambo	0.88(0.57,1.34)	0.23	0.55(0.18,1.67)	0.51	0.95(0.60,1.49)	0.82	0.75(0.60,1.49)	0.83
Uvinza	1.09(0.14,8.63)	0.94	0.57(0.05, 6.09)	0.64	0.83(0.73,0.99)	0.01	0.81(0.73,0.99)	0.03
Uyui	1.11(0.12,4.61)	0.32	1.17(0.13,4.65)	0.39	0.08(0.07,1.09)	0.12	0.28(0.07,1.49)	0.17

Supplementary material 2b. Crude and Adjusted Odds ratio between neoplasm of breast and head and neck diagnosed cases and districts

Variables	Malignant neoplasm of the breast cOR(95%CI)	P-value	aOR(95%CI)	P-value	Malignant neoplasm of head and neck cOR(95%CI)	P-value	aOR(95%CI)	P-value
**District**								
Bariadi	1.14(0.62,2.09)	0.68	0.60(0.31,1.20)	0.16	1.28(0.68, 2.41)	0.44	1.28(0.68,2.41)	0.45
Biharamulo	0.84(0.26,2.74)	0.77	0.53(0.11, 2.67)	0.44	1.43(0.50, 4.06)	0.50	1.43(0.50,4.06)	0.42
Buhigwe	9.8(0.61,157)	0.11	11.0(0.29,4.29)	0.19	0.19(0.03,0.28)	0.02	0.08(0.07,0.09)	0.01
Bukoba	0.96(0.46,1.99)	0.91	0.21(0.02,2.03)	0.18	0.41(0.13,1.31)	0.13	0.41(0.13,1.31)	0.13
Bukombe	0.47(0.06, 3.47)	0.43	0.46(0.19,1.13)	0.09	1.12(0.51,2.45)	0.78	0.58(0.08,4.29)	0.59
Bunda	0.76(0.33,1.76)	0.52	0.69(0.11,4.35)	0.69	2.92(1.09,7.79)	0.03	1.12(0.51,2.45)	0.78
Busega	0.82(0.19,3.47)	0.78	0.35(0.14,0.84)	0.02	1.17(0.37,3.86)	0.79	2.9(1.09,7.79)	0.03
Butiama	1.70(0.65,4.42)	0.28	1.60(0.29,4.25)	0.15	1.46(0.50, 4.05)	0.40	1.17(0.36,3.87)	0.79
Chato	0.82(0.19, 0.97)	0.01	1.50(0.22,4.26)	0.34	0.81(0.19,3.39)	0.77	0.81(0.19,3.39)	0.77
Geita	0.43(0.19,0.97)	0.04	0.75(0.43,1.29)	0.12	1.24(0.69,2.23)	0.47	1.24(0.69,2.23)	0.47
Igunga	0.89(0.21,3.81)	0.45	0.55(0.12, 2.53)	0.44	0.52(0.07,3.90)	0.53	0.52(0.07,3.90)	0.53
Ilemela	1.91(1.44,2.53)	<0.001	0.25(0.03,2.40)	0.26	0.78(0.53,1.15)	0.21	0.78(0.53,1.15)	0.23
Itilima	0.38(0.05,2.78)	0.33	0.42(0.18,0.96)	0.04	0.46(0.06,3.43)	0.45	0.46(0.06,3.4)	0.45
Kahama	1.43(1.36,1.73)	0.03	1.34(1.12,4.68)	0.03	0.99(0.45,2.15)	0.97	0.99(0.45,2.15)	0.97
Kakonko	0.58(0.07,4.34)	0.59	0.60(0.06,6.54)	0.68	1.21(0.15,9.49)	0.86	1.21(0.15,9.49)	0.65
Kaliua	0.53(0.07,1.31)	0.34	1.04(0.19,5.49)	0.96	2.44(0.70,8.47)	0.16	2.44(0.70,8.47)	0.19
Karagwe	1.13(0.34,3.76)	0.83	1.41(0.38,5.31)	0.61	1.40(0.42,4.66)	0.58	1.41(0.42,4.68)	0.57
Kasulu	2.09(0.97,4.52)	0.06	2.0(0.91,4.54)	0.08	1.16(1.01,3.25)	0.02	1.18(1.11,3.25)	0.01
Kibondo	1.83(0.69,4.78)	0.22	1.73(0.38,7.77)	0.71	1.26(0.38,4.15)	0.71	1.35(0.38,4.15)	0.75
Kigoma	2.11(0.60,7.39)	0.24	1.72(0.41,7.14)	0.75	0.76(0.09, 5.72)	0.79	0.73(0.19,5.72)	0.77
Kishapu	0.59(0.14,2.48)	0.47	0.26(0.04,1.57)	0.14	1.14(0.35,3.74)	0.83	1.16(0.32,3.74)	0.87
Kwimba	0.16(0.02,1.19)	0.07	0.09(0.01,0.93)	0.04	0.41(0.10, 1.70)	0.22	0.41(0.10,1.70)	0.27
Kyerwa	1.64(0.19,13.6)	0.65	0.70(0.06,8.47)	0.78	1.21(0.36,4.19)	0.67	1.25(0.38,4.17)	0.69

***Supplementary material 3a*.** Crude and Adjusted Odds ratio between neoplasm of oesophagus and non-Hodgkin lymphomas diagnosed cases and districts

	**Malignant neoplasm of the oesophagus**				**Non-Hodgkin lymphomas**			
**Variables**	**cOR(95%CI)**	**P-value**	**aOR(95%CI)**	**P-value**	**cOR(95%CI)**	**P-value**	**aOR(95%CI)**	**P-value**
**District**								
Bariadi	1.34(1.11,2.53)	0.02	1.35(1.22,1.87)	0.01	1.27(0.68, 2.67)	0.47	1.56(0.64,2.44)	0.43
Biharamulo	0.67(0.24,2.71)	0.76	0.55(0.14, 2.68)	0.47	1.67(0.50, 3.06)	0.56	1.47(0.56,3.06)	0.45
Buhigwe	1.85(0.63,1.57)	0.16	1.30(0.24,2.27)	0.24	0.16(0.03,0.26)	0.01	0.18(0.17,0.09)	0.01
Bukoba	0.67(0.45,1.97)	0.87	0.25(0.12,2.03)	0.17	0.48(0.13,1.31)	0.17	0.47(0.23,1.31)	0.18
Bukombe	0.44(0.15, 2.46)	0.34	0.35(0.21,1.13)	0.34	1.19(0.51,2.43)	0.74	0.38(0.08,4.45)	0.57
Bunda	0.66(0.39,1.76)	0.54	0.67(0.11,4.37)	0.87	2.95(1.05,7.79)	0.03	1.18(0.11,2.45)	0.75
Busega	0.78(0.21,3.42)	0.56	0.35(0.14,0.84)	0.02	1.18(1.07,3.87)	0.03	2.30(1.09,7.77)	0.03
Butiama	1.69(0.57,2.47)	0.45	1.73(0.24,4.27)	0.18	1.47(0.53, 4.05)	0.43	1.16(0.26,3.89)	0.76
Chato	0.84(0.14, 0.96)	0.01	1.43(0.22,4.27)	0.35	0.84(0.19,3.35)	0.75	0.82(0.14,3.39)	0.75
Geita	1.43(1.16,1.97)	0.04	1.75(1.43,1.76)	0.02	1.26(0.63,2.23)	0.43	1.27(0.69,2.29)	0.41
Igunga	0.89(0.24,3.87)	0.46	0.53(0.12, 2.58)	0.48	0.58(0.07,3.96)	0.56	0.55(0.17,3.90)	0.52
Ilemela	1.91(0.44,2.53)	0.41	1.34(1.12,2.40)	0.03	0.79(0.52,1.15)	0.41	0.79(0.53,1.18)	0.28
Itilima	0.38(0.05,2.78)	0.33	0.42(0.18,0.96)	0.04	0.47(0.06,3.54)	0.49	0.44(0.16,3.45)	0.42
Kahama	0.79(0.36,1.73)	0.56	1.34(0.42,4.68)	0.53	0.98(0.44,2.15)	0.57	0.96(0.45,2.13)	0.98
Kakonko	0.58(0.05,2.34)	0.57	0.65(0.16,3.54)	0.64	1.25(0.15,6.47)	0.82	1.26(0.14,9.49)	0.65
Kaliua	0.54(0.17,1.35)	0.33	1.24(0.19,4.49)	0.94	2.46(0.73,8.47)	0.15	2.48(0.70,7.47)	0.16
Karagwe	1.43(0.34,3.73)	0.84	1.32(0.38,3.37)	0.56	1.43(0.42,4.68)	0.56	1.44(0.42,5.68)	0.59
Kasulu	2.02(0.97,4.52)	0.12	2.0(0.91,4.53)	0.07	1.15(0.44,3.25)	0.77	1.19(0.21,3.25)	0.38
Kibondo	1.82(0.63,4.78)	0.22	1.73(0.38,7.77)	0.74	1.29(0.38,4.17)	0.86	1.34(0.38,4.16)	0.74
Kigoma	2.11(0.60,7.39)	0.25	1.72(0.41,7.14)	0.77	0.74(0.29, 5.72)	0.56	0.78(0.19,5.72)	0.97
Kishapu	0.45(0.17,2.48)	0.47	0.26(0.04,1.57)	0.13	1.16(0.37,3.74)	0.84	1.12(0.32,3.74)	0.85
Kwimba	0.14(0.02,1.19)	0.04	0.09(0.01,0.2)	0.04	0.49(0.10, 1.73)	0.29	0.46(0.11,1.73)	0.26
Kyerwa	1.64(0.19,13.6)	0.66	0.70(0.06,8.47)	0.74	1.28(0.34,4.19)	0.65	1.28(0.38,4.17)	0.64

Supplementary material 3b. Crude and Adjusted Odds ratio between neoplasm of oesophagus and non-Hodgkin lymphomas diagnosed cases and districts

Variables	Malignant neoplasm of the oesophagus cOR(95%CI)	P-value	aOR(95%CI)	P-value	Non-Hodgkin lymphomas cOR(95%CI)	P-value	aOR(95%CI)	P-value
**District**								
Magu	0.64(0.24,1.51)	0.28	0.48(0.18,1.29)	0.14	0.78(0.30,1.85)	0.53	0.77(0.32,1.88)	0.56
Maswa	0.28(0.35,2.76)	0.40	0.58(0.13,2.56)	0.08	0.56(0.13,2.38)	0.44	0.55(0.12,2.38)	0.43
Mbogwe	0.63(0.17,3.06)	0.26	1.15(0.32,3.74)	0.81	2.25(0.73,6.41)	0.39	2.26(0.73,8.49)	0.17
Meatu	0.76(0.09,5.78)	0.19	0.42(0.07, 2.52)	0.34	0.46(0.16,3.18)	0.45	0.44(0.16,3.18)	0.43
Missenyi	0.74(0.15,3.04)	0.44	1.24(0.10,14.4)	0.18	0.92(0.12,7.34)	0.65	0.92(0.12,7.18)	0.89
Misungwi	1.42(0.56,3.73)	0.25	1.59(0.36,6.96)	0.51	1.25(0.35,2.63)	0.66	1.27(0.15,2.63)	0.66
Muleba	0.16(0.17,3.05)	0.65	0.27(0.09,0.81)	0.08	1.11(0.31,2.29)	0.94	1.29(0.31,3.43)	0.95
Musoma	0.47(0.16,1.23)	0.14	0.45(0.17,1.25)	0.17	2.27(1.16,4.06)	0.01	2.21(1.17,4.06)	0.01
Ngara	1.69(0.37,2.35)	0.58	0.89(0.13, 5.73)	0.85	0.59(0.03,3.44)	0.45	0.56(0.03,2.35)	0.42
Nzega	0.48(0.10,1.76)	0.27	0.65(0.07,1.28)	0.13	1.67(0.72,4.04)	0.21	1.73(0.73,4.04)	0.27
Nyamagana	1.34(1.03,1.77)	0.02	0.86(0.58,1.21)	0.37	1.18(0.79,1.57)	0.53	1.14(0.79,1.56)	0.49
Nyang'hwale	1.35(0.31,2.35)	0.58	1.46(0.51,3.34)	0.57	0.33(0.17,0.99)	0.01	0.85(0.27,0.99)	0.01
Rorya	0.37(0.08,1.39)	0.17	0.23(0.05,0.87)	0.03	0.85(0.31,2.36)	0.78	0.86(0.31,2.36)	0.72
Sengerema	1.12(0.84,1.65)	0.53	1.24(0.27, 3.97)	0.94	0.72(0.41,1.20)	0.23	0.75(0.54,1.20)	0.26
Serengeti	1.17(0.33,3.76)	0.56	1.36(0.22, 3.56)	0.53	0.53(0.06, 3.17)	0.47	0.48(0.26,3.17)	0.33
Shinyanga	0.95(0.28,1.43)	0.23	0.35(0.17,0.67)	0.01	1.14(0.54,1.85)	0.91	1.04(0.54,1.85)	0.76
Sikonge	1.49(0.41,2.78)	0.19	1.45(0.21,2.65)	0.17	1.66(0.37,7.18)	0.53	1.57(1.33,7.16)	0.02
Tabora municipal	0.56(0.22,1.65)	0.33	0.47(0.18,1.26)	0.18	1.25(0.34,2.95)	0.94	1.25(0.35,2.95)	0.45
Tarime	1.45(0.43,2.76)	0.35	0.32(0.11,1.94)	0.04	0.53(0.37,2.39)	0.85	0.94(0.37,2.37)	0.87
Ujiji MC	4.44(1.35,14.3)	0.01	4.48(1.08,18.5)	0.04	1.21(0.14,7.79)	0.96	1.01(0.12,7.79)	0.34
Ukelewe	0.96(0.32,2.56)	0.82	0.65(0.14,2.69)	0.49	2.16(0.65,4.84)	0.08	2.65(0.95,4.83)	0.57
Urambo	0.83(0.57,1.34)	0.23	0.76(0.18,1.78)	0.51	0.85(0.60,1.47)	0.86	0.75(0.65,1.49)	0.84
Uvinza	1.23(0.14,3.63)	0.92	0.56(0.15, 4.09)	0.64	0.87(0.74,0.99)	0.01	0.81(0.73,0.94)	0.03
Uyui	1.34(0.12,4.61)	0.39	1.42(0.13,3.65)	0.39	0.28(0.07,1.08)	0.14	0.28(0.17,1.49)	0.19
